# Male infertility with muscle weakness: a point of view

**DOI:** 10.1097/MS9.0000000000001147

**Published:** 2023-08-14

**Authors:** Naram Khalayli, Bassel Achmeh, Khalil Ali, Aghiad Aziz, Maysoun Kudsi

**Affiliations:** a Psychiatry Department; b Faculty of Medicine; c Rheumatology Department, Faculty of Medicine, Damascus University, Damascus, Syria

**Keywords:** azoospermia, inherited diseases, male infertility, muscle weakness, myotonic dystrophy

## Abstract

**Introduction and importance:**

The most common causes of infertility are idiopathic spermatogenetic disorders, occurring in multiple reproductive or systemic diseases. The underlying genetic disorders influence the treatment and transmission of the disease to the offspring.

**Case presentation:**

A 32-year-old Syrian male, married for 6 years, presented with primary infertility. The patient had a history of muscle dystrophy for 12 years. He had no previous medical or drug addiction or family history. He had gynecomastia. Semen analysis revealed oligospermia in the patient. Follicle-stimulating hormone was elevated. Gene analysis could not be done due to funding issues. The percutaneous testicular biopsy revealed hypospermatogenesis, atrophy, and marked hyalinization of the seminiferous tubules. Electromyography of the upper extremities demonstrated myotonic discharges, with a waxing–waning frequency, amplitude, and a characteristic ‘engine revving’ sound.

**Clinical discussion:**

Myotonic dystrophy (MD) is an autosomal dominant inheritance disease with adult onset. Muscle weakness is the predominant presenting feature, with early involvement of the distal limbs and neck muscles and a characteristic facial appearance.

Systemic clinical manifestations may include cardiac conduction defects, cataracts, insulin resistance and diabetes, testicular atrophy with impaired spermatogenesis, and others. Testicular biopsy findings are specific. To our knowledge, this is the first case of male infertility associated with MD in Syria. However, there are no data on the prevalence of myotonic dystrophy type 1 (MD1) in Syria.

**Conclusion:**

The practicing physician should keep in mind the frequent association between MD and infertility.

## Introduction and importance

Highlights

- Spermatogenic disorders are the most frequent cause of male infertility.
- Correlation between muscular dystrophy and hypogonadism.
- Evidence of fibrosis on testicular biopsy.
- Other exploratory treatment options for infertility.
- Sperm extraction and injections into the female reproductive system.

Male infertility (MI) is a prevalent issue in ~50% of couples seeking assisted reproductive treatments^1^. The primary cause of this condition is often idiopathic spermatogenetic disorders, specifically oligozoospermia, which is characterized by a low sperm count. It can be associated with various reproductive or systemic diseases^2^. A comprehensive evaluation of medical history and physical examination can help identify potential treatable causes of oligozoospermia, although in certain cases, treatment options may be limited^3^. It is essential to consider underlying genetic disorders as they can impact treatment outcomes and the potential transmission of the disease to future generations^4^. We discuss a case of a male patient with muscle dystrophy associated with infertility.

## Case presentation

A 32-year-old Syrian male, married for 6 years, presented to the clinic seeking medical advice for rheumatologic consultation for primary infertility, despite engaging in regular sexual activities. He had normal libido, preserved spontaneous erections, and satisfactory tumescence. He had a history of muscle dystrophy for 12 years. He had no previous medical history, drug addiction, or family history.

He appeared healthy, his height was 162 cm and his body weight was 75 kg. He had visible atrophy of the masseter, temporalis, and neck muscles, with movements of the neck against resistance; the sternocleidomastoid muscle was found atrophied bilaterally.

**Figure 1:**
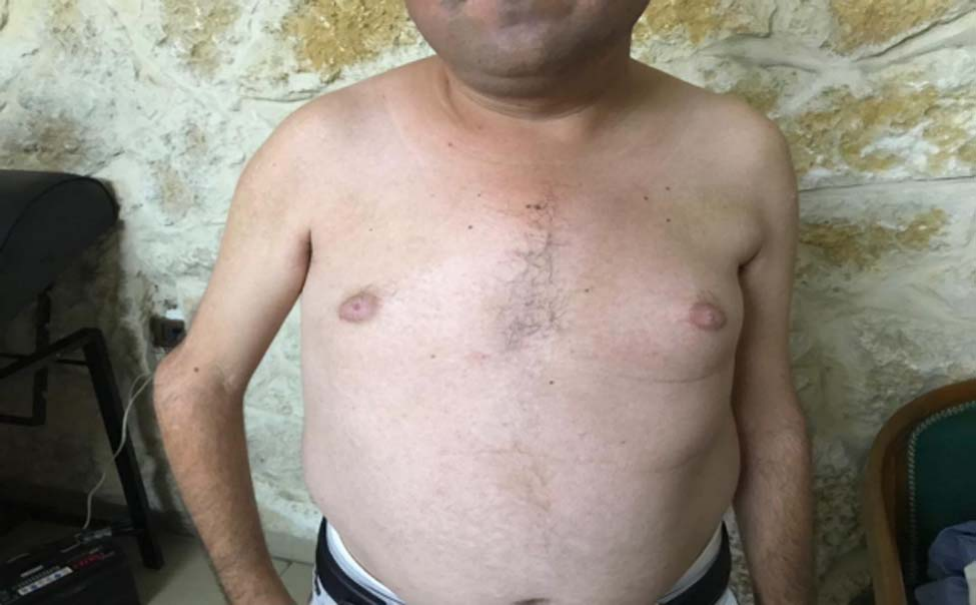
Anterior inspection for muscle wasting

**Figure 2:**
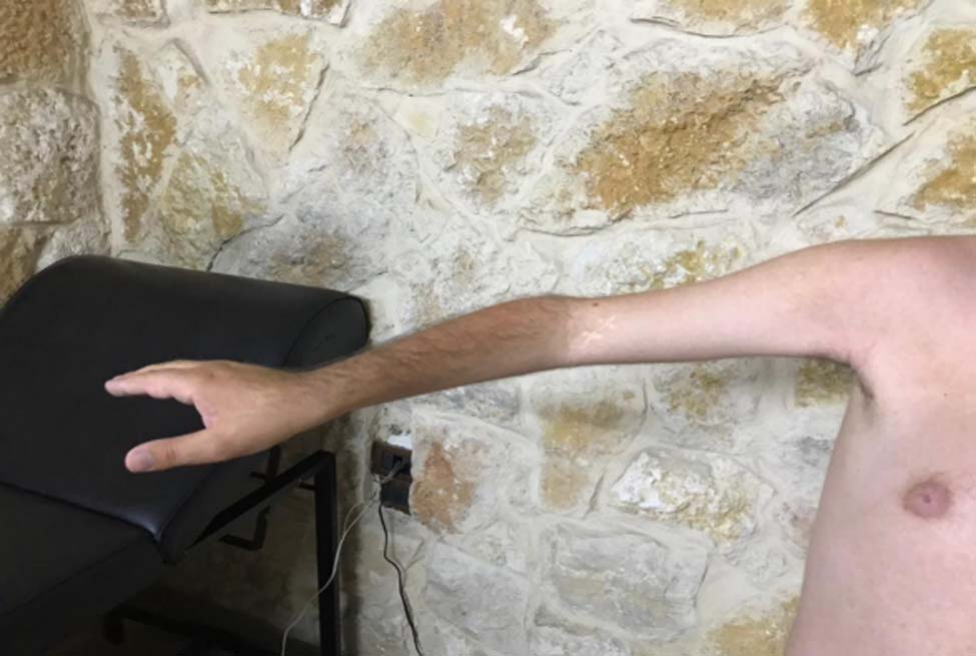
Muscle wasting on the right arm

**Figure 3:**
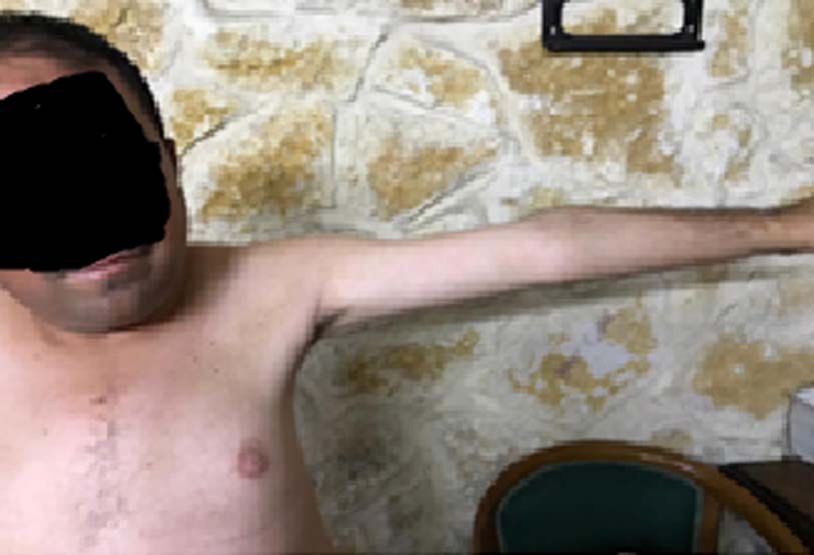
Muscle wasting on the left arm

On neurological examination, he had muscle weakness only in the upper extremities, leading to difficulty releasing objects from his handgrip and lifting his arms up, developing for 12 years. He had gynecomastia (Figs 1–3).

**Figure 4:**
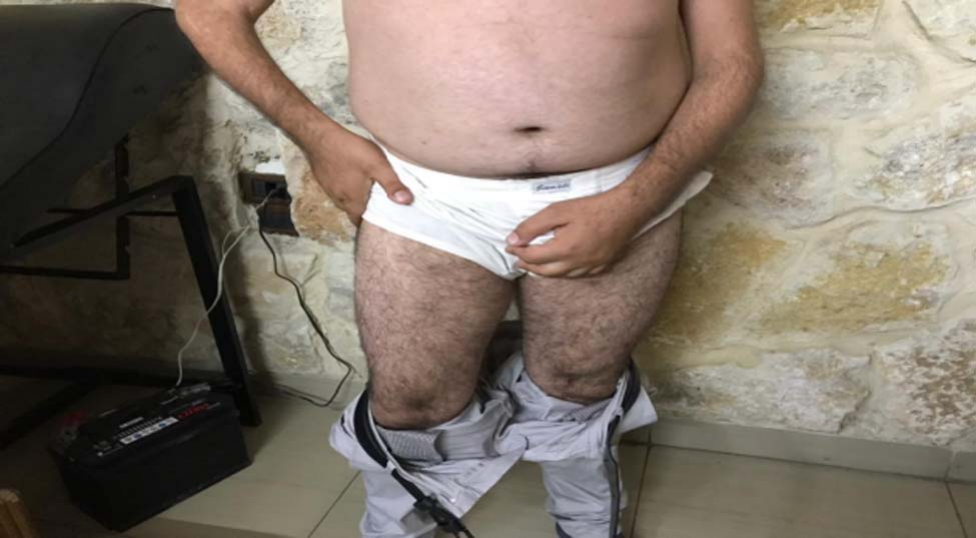
Spared leg muscles from muscular atrophy

The lower extremity muscles were normal (Fig. 4) on neurological examination.

Secondary sexual characteristics were normal, and he was adequately virilized, but the testes were soft and atrophied in size, measuring 4 ml on the left and 4.2 ml on the right. Bilaterally, vas deferens and epididymides were normal on palpation. Monthly separated semen analysis in three samples showed oligospermia (sperm count <5×10^6^/ml, normal: 15–259×10^6^/ml), average ejaculate volume of 1.5 ml, and pH between 7.3 and 7.5 with normal seminal fluid fructose, respectively.

Early morning serum testosterone, luteinizing hormone (LH), aldolase, and serum creatine kinase were found to be within normal ranges. Follicle-stimulating hormone (FSH) was elevated at 21.3 mIU/ml (1.1–13.5). The immune profile was negative. Gene analysis could not be done due to fund issues.

The percutaneous testicular biopsy revealed hypospermatogenesis, atrophy, and marked hyalinization of the seminiferous tubules.

Electromyography of the upper extremities demonstrated myotonic discharges with a waxing–waning frequency, amplitude, and a characteristic ‘engine revving’ sound, while the electromyography of the lower extremities was normal.

In light of this evidence, a diagnosis of myotonic dystrophy (MD) was made.

This case report has been reported in line with the Surgical CAse REport (SCARE) Criteria^5^

## Clinical discussion

MD is an autosomal dominant inheritance disease that typically manifests in adulthood^6^. Our patient, a 32-year-old individual, has been experiencing symptoms of MD for the past 12 years. There are two types of MD: type 1, which is characterized by an unstable repeat expansion of CTG (cytosine–thymine–guanine) in the 3′-untranslated region of the dystrophia myotonica protein kinase gene, and type 2, which is caused by a defect in the CCHC-type zinc finger, nucleic acid-binding protein gene^7^.

Muscle weakness is the primary symptom in most patients, with early involvement of distal limbs and neck muscles, including the sternocleidomastoids^8^. These individuals often have a distinctive facial appearance^7^, which is also seen in our patient. MD can have systemic clinical manifestations, such as cardiac conduction defects, cataracts, insulin resistance, diabetes, difficulty in swallowing, gastrointestinal motility issues, and testicular atrophy leading to impaired spermatogenesis^8^. In our patient’s case, he presented only with testicular atrophy.

MI is a well-recognized consequence of MD, although it is often overlooked in clinical practice. Approximately 80% of young to middle-aged males with MD experience primary hypogonadism^7–9^. These individuals tend to have isolated impairment of sperm production or function, and testicular biopsy often reveals atrophy with hyalinization and fibrosis of the seminiferous tubules^7–10^, which was consistent with our case. High serum gonadotropin levels, particularly elevated FSH levels compared to LH levels, are commonly found in these patients^7^. Such elevated FSH levels can lead to an altered estradiol-to-testosterone ratio and potentially result in gynecomastia^11^, as observed in our patient.

Various treatment options are available for MI associated with MD, including testicular sperm extraction or aspiration and microsurgical epididymal sperm aspiration with intra-cytoplasmic sperm injection^12^.

To our knowledge, this is the first case of MI associated with MD1 in Syria. Although MI in Arabs is fairly common, there is a dearth in published reports of genetic epidemiology of MI, no data on the prevalence of MD1 in Syria^13^. The limitations in our case were the genetic studies, as many of them are not available in Syria, in addition to the higher costs.

A significant number of cases of idiopathic azoospermia may have a genetic etiology. Consequently, some of these patients may harbor larger trinucleotide repeat alleles, which can contribute to an elevated risk of heritable diseases in their progeny^6,7,14,15^.

It is imperative to establish a correct diagnosis of the underlying condition and provide genetic counseling to prevent the transmission of these heritable diseases to future generations^16^.

## Conclusion

The practicing physician should keep in mind the frequent association between MD and infertility.

## Ethical approval

The Ethical Committee of Faculty of Medicine number WQA; 23143, 2023 had approved this case report.

## Consent

Written informed consent was obtained from the patient for the publication of this case report and accompanying images. A copy of the written consent is available for review by the Editor-in-Chief of this journal on request.

## Sources of funding

No funding.

## Author contribution

N.K.: data curation and writing – review and editing; B.A.: conceptualization and writing – original draft, review, and editing; K.A.: writing – review and editing; A.A.: resources; M.K.: supervision and writing – review and editing.

## Conflicts of interest disclosure

The authors declares that they have no conflicts of interest.

## Research registration unique identifying number (UIN)

1. Name of the registry: Researchregistry9109.
2. Unique identifying number or registration ID: 9109.
3. Hyperlink to your specific registration (must be publicly accessible and will be checked): https://www.researchregistry.com/browse-the-registry#home/.

## Guarantor

Maysoun Kudsi (Professor of Rheumatology Department, Faculty of Medicine, Damascus University).

## Data availability statement

Data are available.

## Provenance and peer review

Not commissioned, externally peer-reviewed.
